# From Exposure to Biomarker: Cumulative Tobacco Burden and Integrated Multiomics Signatures of High Tumor Mutational Burden in Lung Adenocarcinoma—A Secondary Analysis of the Cancer Genome Atlas

**DOI:** 10.1155/humu/1906706

**Published:** 2026-07-08

**Authors:** Jin Wei, Qin Yangsong, Ruan Hongjia, Zhu Bingqiang, Yang Conggao

**Affiliations:** ^1^ Kunming Ecological Environmental Engineering Assessment, Kunming, China; ^2^ Faculty of Environmental Science and Engineering, Kunming University of Science and Technology, Kunming, China, kmust.edu.cn

**Keywords:** biomarker translation, cBioPortal, elastic net, lung adenocarcinoma, multiomics, TCGA, tobacco exposure, tumor mutational burden

## Abstract

Environmental exposures are upstream determinants of molecular variation, yet exposure‐to‐biomarker gradients remain insufficiently quantified in harmonized multiomics cancer cohorts. Using TCGA lung adenocarcinoma as a model, we evaluated cumulative tobacco exposure and smoking history as determinants of variant‐derived biomarkers and tested whether integrating clinical, genomic, transcriptomic, and proteomic data improves identification of high tumor mutational burden (TMB). This secondary analysis used public cBioPortal‐linked TCGA data. Among 522 patients with clinical and molecular annotations, 302 ever‐smokers with nonmissing pack‐years, TMB, and covariates comprised the primary adjusted logistic‐regression cohort, and 250 cases had matched multiomics data for prediction modeling. Higher cumulative tobacco exposure was associated with greater odds of high TMB: Compared with pack‐year tertile 1, tertile 3 had an adjusted odds ratio of 2.28 (95% CI: 1.18, 4.41; *p* trend = 0.013), and each 10 pack‐year increment was associated with an odds ratio of 1.12 (95% CI: 1.02, 1.23). Smoking categories also showed strong gradients for TMB, total nonsynonymous mutation counts, and C > A substitution fraction. Current smoking was positively associated with TP53 mutation and inversely associated with EGFR mutation relative to never‐smoking. High‐TMB tumors showed 201 differentially expressed transcripts and 15 differentially abundant proteins. Driver‐augmented models discriminated high TMB better than broader multiomics models, although integrated scores retained prognostic relevance. These findings support exposure‐aware biomarker development in lung adenocarcinoma. High TMB was defined as a cohort‐specific top‐quartile analytic endpoint rather than a universal clinical threshold. The findings support biomarker interpretation and hypothesis generation, not direct immunotherapy‐response prediction or a clinically deployable model.

## 1. Introduction

Lung adenocarcinoma is shaped by both inherited susceptibility and environmental exposures, with tobacco smoking remaining its most prominent modifiable determinant [1–3]. In addition to increasing cancer incidence, smoking imprints the tumor genome with reproducible mutational patterns and influences the prevalence of recurrent driver events, immune contexture, and treatment response [4–6]. These features make tobacco exposure an especially informative environmental axis for studying how upstream exposures become clinically interpretable molecular biomarkers. Mechanistically, tobacco‐related carcinogens generate DNA damage and mutational signatures enriched for C > A substitutions [4, 7–10]; accumulation of such coding‐region mutations provides a direct bridge from smoking exposure to elevated nonsynonymous mutation burden and TMB.

The TCGA lung adenocarcinoma project established lung adenocarcinoma as a paradigmatic multiomics disease, integrating somatic variation, copy‐number changes, transcriptomics, methylation, and proteomic data in a single harmonized resource [11–13]. Public portals such as cBioPortal now allow secondary analyses that connect curated clinical annotations, including smoking history and cumulative pack‐years, to variant and biomarker layers across the same tumors [14–16]. TCGA LUAD is an appropriate model system for this exposure‐to‐biomarker question because LUAD includes etiologically heterogeneous smoker and never‐smoker tumors and provides matched clinical, genomic, transcriptomic, and proteomic layers [11–19] within the same disease context.

Among clinically translatable biomarkers, TMB remains important because it condenses somatic variation into a summary measure linked to immunotherapy response and molecular phenotype in multiple solid tumors [7–10]. Yet TMB should not be interpreted in isolation. Exposure‐associated TMB is embedded within broader mutational classes, recurrent driver alterations, transcriptomic programs, and proteomic states that may either reinforce or complicate its translational meaning. Because high TMB carries clinical connotations in immunotherapy settings, the present study uses high TMB strictly as a cohort‐specific top‐quartile analytic biomarker endpoint [7–10], not as a universal clinical threshold or as a direct predictor of immunotherapy response.

We therefore selected the TCGA lung adenocarcinoma cohort for a secondary analysis centered on an explicitly environmental determinant‐cumulative tobacco burden‐and evaluated its relationship to a variant‐derived biomarker, high TMB. Following a structured analytic framework based on staged descriptive, regression, and biomarker analyses, we further extended the approach with spline modeling, differential multiomics profiling, cross‐validated elastic‐net prediction, and survival analyses to better characterize the exposure‐to‐biomarker pathway relevant to clinical translation. What remained unresolved after prior TCGA and smoking‐related LUAD studies was whether categorical smoking status and cumulative pack‐years provide complementary exposure information and how those exposure measures map across variant‐derived, transcriptomic, and proteomic biomarker layers in the same tumors. The present analysis adds an explicit exposure‐to‐biomarker framework by jointly evaluating smoking status, cumulative pack‐years, high‐TMB classification, other variant endpoints, differential multiomics correlates, internal prediction, and survival patterns. In this manuscript, exposure‐aware biomarker development means that biomarker definition, modeling, and interpretation explicitly account for upstream exposure information such as smoking status and cumulative pack‐years.

## 2. Methods

### 2.1. Study Population and Design

This study was a secondary analysis of publicly available deidentified TCGA lung adenocarcinoma data accessed through cBioPortal [17–19]. We linked patient‐level clinical annotations from the TCGA lung adenocarcinoma study to sample‐level PanCancer Atlas molecular annotations to obtain smoking history, cumulative pack‐years, survival, recurrent somatic alterations, TMB, and ancillary biomarker metrics. When multiple samples were present for the same patient, primary tumor samples were prioritized. No imputation of missing values was performed; each analysis was run in the subset with available data for the required variables.

The full linked cohort contained 522 patients with clinical and molecular annotations. Of these, 508 had documented smoking‐history codes, 356 had nonmissing cumulative pack‐years, 514 had sample‐level TMB annotations, and 250 had matched clinical, recurrent‐driver, transcriptomic, proteomic, and TMB data suitable for integrated modeling. Because pack‐years are principally interpretable among ever‐smokers and because the smoking‐history distribution introduced sparse cells among never‐smoking cases in adjusted pack‐year models, the primary logistic analyses were restricted to ever‐smokers with nonmissing pack‐years, TMB, and covariates (*n* = 302). These analytic subsets are overlapping rather than strictly nested because each endpoint or model required a different set of available clinical, molecular, and omics variables; their derivation is summarized in Figure S4.

### 2.2. Assessment of Tobacco Exposure

Smoking history was derived from the TCGA smoking‐history indicator and analyzed in its harmonized categories: lifelong nonsmoker, current smoker, former smoker > 15 years, former smoker ≤ 15 years, and former smoker with unspecified cessation interval. For specific models, categories were collapsed into never, current, and former smoking. Cumulative tobacco exposure was assessed using the recorded smoking pack‐years variable and analyzed continuously (per 10 pack‐year increment), by spline terms, and in tertiles. In the primary adjusted pack‐year cohort, tertile ranges were 0.15–28, 29–48, and 50–154 pack‐years. Smoking‐history category and pack‐years were retained as complementary exposure measures: Category captures current/former/never status and cessation timing, whereas pack‐years summarizes cumulative dose. Continuous, tertile‐based, and spline analyses were used to distinguish approximately linear dose effects from possible threshold or upper tail patterns.

### 2.3. Somatic Variant and Multiomics Characterization

TMB was derived from the PanCancer Atlas sample‐level nonsynonymous mutation burden annotation and treated as a continuous measure and as a dichotomous biomarker endpoint. Additional molecular summaries included sample‐level aneuploidy score and a substitution‐based C > A fraction computed from nonsilent single‐nucleotide variants because tobacco‐associated mutagenesis is enriched for this substitution class [4]. Recurrent driver alterations were summarized as patient‐level binary indicators for TP53, KRAS, KEAP1, STK11, EGFR, ALK, NF1, RBM10, SMARCA4, and BRAF.

Transcriptomic analyses used mRNA *z* scores, whereas proteomic analyses used RPPA *z* scores. For differential analyses, feature‐wise comparisons contrasted high‐TMB and nonhigh‐TMB tumors. For prediction modeling, the multiomics design matrix included clinical variables, recurrent driver events, 60 high‐variance transcripts, and 25 high‐variance proteins, followed by foldwise univariate feature selection within the modeling pipeline.

### 2.4. Definition of Biomarker Endpoints

The primary biomarker endpoint was high TMB, defined a priori as the cohort‐specific top quartile of sample‐level TMB. Secondary biomarker endpoints were high aneuploidy, high C > A fraction, and binary recurrent driver alterations. An integrated multiomics score was defined as the out‐of‐fold predicted probability of high TMB from the combined clinical plus variant plus multiomics elastic‐net model. This definition was chosen to preserve internal contrast and statistical power in TCGA LUAD and should be interpreted as an analytic endpoint for this cohort, not as a clinically validated cutoff.

### 2.5. Covariates

Adjusted models considered age (≥ 65 years), sex, race (White compared with non‐White), stage (I–II compared with III–IV), smoking status (current compared with former, in pack‐year models), and history of other malignancy. These covariates were selected because they were clinically interpretable, available in the public dataset, and potentially related to both tobacco exposure and biomarker distributions.

### 2.6. Statistical Analysis

Continuous variables are presented as means and SDs, and categorical variables as *n* (%). The primary association between cumulative tobacco exposure and high TMB was evaluated using logistic‐regression models. Model 1 was adjusted for age, sex, race, and stage. Model 2 was further adjusted for current‐ versus former‐smoking status and history of other malignancy. Linear trend across pack‐year tertiles was assessed using the tertile median as a continuous term.

Differences in molecular summary metrics across smoking‐history categories were tested using adjusted linear models. Differential transcriptomic and proteomic analyses compared high‐TMB with nonhigh‐TMB tumors using feature‐wise Welch tests, with Benjamini–Hochberg false discovery rate (FDR) control [20]. To allow flexible dose‐response assessment, pack‐years was also modeled with spline terms. Predictive modeling used five‐fold outer cross‐validation with elastic‐net logistic regression [21]; the multiomics model additionally used foldwise univariate feature selection after unsupervised variance filtering. Model performance was summarized by out‐of‐fold AUROC with bootstrap 95% CIs. Exploratory overall‐survival analyses used Cox proportional hazards models adjusted for age, sex, and stage [22]. All tests were two‐sided and *p* < 0.05 was considered statistically significant unless FDR adjustment was specified. Elastic‐net logistic regression was selected because the prediction cohort had a moderate sample size relative to many correlated genomic, transcriptomic, and proteomic candidate predictors; the combined L1/L2 penalty performs shrinkage and variable selection while retaining groups of correlated features, reducing overfitting in cross‐validation.

## 3. Results

### 3.1. Characteristics of the Study Population According to Cumulative Tobacco Exposure

Table 1 shows the primary adjusted pack‐year cohort. The tertile distribution was unbalanced after complete‐case restriction because missing covariates and tied pack‐year values reduced the highest exposure stratum more than the lower strata. Nevertheless, increasing pack‐year tertiles identified clinically distinct tumors. The heaviest exposure tertile contained a higher proportion of males and current smokers, and it also showed progressively higher mean TMB and a larger TP53‐mutant fraction. In contrast, the proportion of EGFR‐mutant tumors remained low across ever‐smoking tertiles.

**Table 1:** Characteristics of the primary adjusted ever‐smoking cohort according to cumulative tobacco exposure tertiles.

Characteristic	Tertile 1 (0.15–28 pack‐years)	Tertile 2 (29–48 pack‐years)	Tertile 3 (50–154 pack‐years)
*n* (%)	127 (42.1)	100 (33.1)	75 (24.8)
Pack‐years, mean (SD)	19.55 (8.55)	42.54 (5.52)	79.01 (23.19)
Age, years	64.19 (11.75)	65.19 (8.98)	66.51 (9.30)
Male sex	43/127 (33.9)	49/100 (49.0)	46/75 (61.3)
White race	105/127 (82.7)	92/100 (92.0)	69/75 (92.0)
Current smoker	22/127 (17.3)	35/100 (35.0)	29/75 (38.7)
Advanced stage (III–IV)	25/127 (19.7)	24/100 (24.0)	13/75 (17.3)
Tumor mutational burden	10.66 (12.64)	11.82 (10.23)	12.61 (9.23)
High TMB (top quartile)	32/127 (25.2)	26/100 (26.0)	31/75 (41.3)
TP53 mutation	54/127 (42.5)	55/100 (55.0)	49/75 (65.3)
KRAS mutation	49/127 (38.6)	28/100 (28.0)	27/75 (36.0)
EGFR mutation	9/127 (7.1)	9/100 (9.0)	4/75 (5.3)

**Figure 1:**
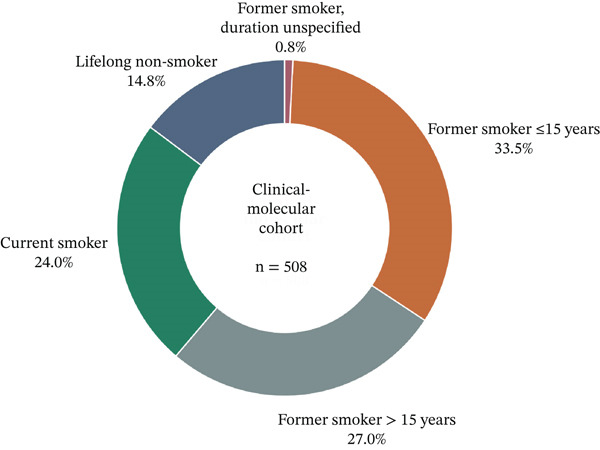
Relative distribution of smoking‐history categories in the clinical‐molecular lung adenocarcinoma cohort. Percentages are based on patients with documented smoking history (*n* = 508).

At the level of smoking history alone, the full clinical‐molecular cohort remained dominated by former and current smoking categories (Figure 1), confirming that TCGA lung adenocarcinoma is well suited for studying environmentally patterned molecular gradients.

### 3.2. Cumulative Tobacco Exposure and the Occurrence of High TMB

The occurrence of high TMB increased from 25.2% in pack‐year tertile 1 to 41.3% in tertile 3. In the minimally adjusted model, tertile 3 showed 2.49‐fold higher odds of high TMB relative to tertile 1. The association remained after additional adjustment for current‐ versus former‐smoking status and prior malignancy (OR: 2.28; 95% CI: 1.18, 4.41; *p* trend = 0.013) (Table 2). When tobacco burden was modeled continuously, each 10 pack‐year increment was associated with higher odds of high TMB in both Model 1 and Model 2. The difference between the modest mean TMB gradient in Table 1 and the larger odds ratio for high TMB indicates that cumulative tobacco exposure mainly increased the probability of falling in the upper tail of the TMB distribution rather than uniformly shifting the entire TMB distribution upward.

**Table 2:** Association between cumulative tobacco exposure and the occurrence of high TMB.

Outcome	Continuous per 10 pack‐years	Tertile 1	Tertile 2	Tertile 3	Adjusted *p* trend
High TMB (n cases/*n* total)	—	32/127	26/100	31/75	—
Model 1	1.13 (1.03, 1.25)	1 (ref)	1.17 (0.63, 2.17)	2.49 (1.30, 4.76)	0.005
Model 2	1.12 (1.02, 1.23)	1 (ref)	1.08 (0.58, 2.04)	2.28 (1.18, 4.41)	0.013

*Note:* High TMB was defined as the top quartile of the sample‐level TMB annotation. Model 1 was adjusted for age, sex, race, and stage. Model 2 was further adjusted for current‐ versus former‐smoking status and history of other malignancy. *p* trend was estimated by entering the median pack‐years value of each tertile as a continuous term.

**Figure 2:**
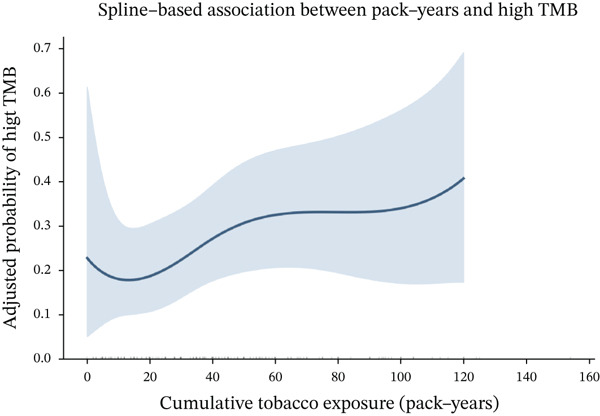
Spline‐based association between cumulative tobacco exposure and the adjusted probability of high TMB in the primary ever‐smoking cohort. The shaded band indicates the 95% confidence interval.

Spline modeling supported a monotonic upward shift in the adjusted probability of high TMB with increasing pack‐years, with the steepest rise occurring beyond approximately 30–40 pack‐years and wider uncertainty only at the extremes of the exposure distribution (Figure 2).

### 3.3. Smoking History and Variant‐Derived Biomarker Metrics

Smoking history categories displayed strong gradients across variant‐derived biomarker summaries (Table 3). Mean TMB was lowest in lifelong never‐smokers (3.07 +/− 3.58) and highest in current smokers and former smokers with unspecified cessation interval, whereas total nonsynonymous mutation counts and the C > A fraction followed the same general pattern. By contrast, aneuploidy varied less strongly across smoking categories and did not retain significance after adjustment.

**Table 3:** Variant‐derived biomarker metrics according to smoking‐history category.

Metric	Current smoker	*F* *o* *r* *m* *e* *r* ≤ 15 *y* *e* *a* *r* *s*	*F* *o* *r* *m* *e* *r* > 15 *y* *e* *a* *r* *s*	Former unspecified	Never smoker	*p* value	FDR *p*
TMB	14.51 ± 13.43	12.07 ± 9.56	7.53 ± 9.02	14.97 ± 8.56	3.07 ± 3.58	< 0.001	< 0.001
Aneuploidy	16.45 ± 7.33	15.96 ± 6.97	13.74 ± 8.42	12.25 ± 6.40	13.79 ± 8.98	0.131	0.131
SNV_TOTAL	596.65 ± 549.13	498.44 ± 395.12	310.36 ± 374.77	627.75 ± 345.51	123.71 ± 149.20	< 0.001	< 0.001
CA_FRAC	0.39 ± 0.12	0.41 ± 0.13	0.35 ± 0.15	0.28 ± 0.16	0.23 ± 0.17	< 0.001	< 0.001

*Note:* Values are means +/− SD. *p* values were derived from adjusted linear models and FDR values used the Benjamini–Hochberg procedure.

**Figure 3:**
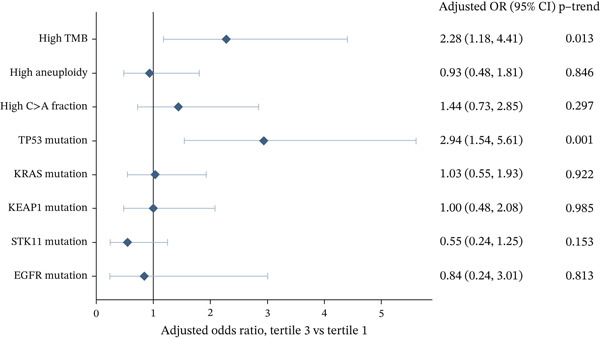
Adjusted odds ratios for selected biomarker endpoints comparing the highest with the lowest tertile of cumulative tobacco exposure. All models were adjusted for age, sex, race, stage, current‐ versus former‐smoking status, and history of other malignancy.

When cumulative pack‐years was used as the exposure metric in endpoint‐specific logistic models, high TMB and TP53 mutation were the outcomes most strongly associated with the heaviest exposure tertile (Figure 3 and Table S1). No material association was observed for high aneuploidy, KRAS mutation, KEAP1 mutation, or EGFR mutation in these pack year–specific comparisons. In the forest plot, high TMB and TP53 mutation stand out as the two endpoints whose point estimates were most clearly displaced above the null for tertile 3 versus tertile 1, whereas the other endpoints clustered closer to no association.

### 3.4. Recurrent Driver Alterations According to Smoking Category

Current smoking was associated with a distinct driver‐mutation architecture when compared with lifelong never‐smoking (Table S2). TP53 mutation was substantially enriched in current smokers (OR: 3.09; 95% CI: 1.59, 6.00), and KRAS mutation was also more frequent (OR: 3.91; 95% CI: 1.51, 10.17). In contrast, EGFR mutation was markedly depleted in current smokers (OR: 0.17; 95% CI: 0.07, 0.41). Associations for KEAP1, STK11, and ALK were directionally consistent with exposure‐related molecular differentiation but did not meet the same significance threshold after FDR control. TP53 is therefore interpreted here as both a smoking‐associated driver‐level event and a correlate of high cumulative mutational burden; these observational analyses do not determine whether TP53 mutation is a cause, consequence, or parallel marker of the high‐TMB state.

### 3.5. Transcriptomic and Proteomic Correlates of High TMB

High‐TMB tumors showed widespread downstream multiomics differences. We identified 201 transcripts and 15 proteins or phosphoproteins associated with high TMB at FDR − adjusted *p* < 0.05. Among transcripts, SRPK2, EPR1, ZNF256, and NCAPD3 were higher in high‐TMB tumors, whereas GKN2, PLA2G10, ITIH2, GLTPD2, EYS, and related features were lower (Table S3). Among proteins, high‐TMB tumors showed higher EIF4EBP1‐pS65, DVL3, Cyclin B1, CD274, TFRC, cleaved CASP7, and MSH6, whereas napsin‐A and progesterone receptor signals were lower (Table S4).

Figure S1 shows the integrated heatmap of selected transcripts and proteins ordered by high‐TMB status and the integrated score. The heatmap confirmed that high‐TMB tumors occupy a coherent transcriptomic‐proteomic state rather than an isolated increase in mutation burden alone. These transcriptomic and proteomic differences should be interpreted as associations with high‐TMB status; the analysis cannot determine whether they are consequences of high TMB, correlates of tobacco exposure, or markers of other tumor biology.

### 3.6. Prediction Performance and Survival Analyses

Cross‐validated discrimination analyses are shown in Table S5 and Figure S2. The clinical model alone produced modest discrimination for high TMB (AUROC: 0.611; 95% CI: 0.528, 0.685). Adding recurrent driver alterations increased AUROC to 0.772 (95% CI: 0.707, 0.831), an absolute gain of 0.162 over the clinical model. The broader multiomics model also improved on the clinical model (AUROC: 0.744; 95% CI: 0.675, 0.807), although it did not exceed the performance of the driver‐augmented model in this internal analysis. Because these were internally cross‐validated models in TCGA without external validation, the performance estimates should be interpreted as exploratory support for biomarker interpretation rather than evidence of a clinically deployable classifier.

Despite its more modest discrimination, the integrated multiomics score retained prognostic relevance. In adjusted Cox models, the highest tertile of the integrated score was associated with worse overall survival relative to the lowest tertile (HR: 1.66; 95% CI: 1.22, 2.27), whereas the per‐SD association was borderline (HR: 1.21; 95% CI: 0.99, 1.48) (Table S6). The corresponding Kaplan–Meier curves are shown in Figure S3. These survival analyses are hypothesis‐generating because treatment details and additional prognostic variables were not comprehensively captured in the public dataset.

## 4. Discussion

### 4.1. Tobacco Exposure and the Translation of Variant‐Derived Biomarkers

This secondary analysis identifies cumulative tobacco burden as a clinically interpretable upstream determinant of a variant‐derived biomarker in lung adenocarcinoma. The main result was robust across categorical, continuous, and spline‐based analyses: Heavier cumulative smoking was associated with a greater likelihood of high TMB [23–25]. These findings are biologically plausible because tobacco smoke generates mutational processes that increase somatic burden and alter the class composition of single‐nucleotide substitutions. The apparent contrast between modest mean TMB differences and larger high‐TMB odds is consistent with exposure‐related enrichment of extreme mutational burden, rather than a simple uniform mean shift.

The observation that the strongest exposure‐linked endpoint was high TMB, rather than aneuploidy, suggests that cumulative smoking is more tightly coupled to mutation‐generating processes than to broad chromosomal instability in this cohort [26–28]. From a clinical‐translation perspective, that distinction matters: It implies that environmental annotation can improve interpretation of TMB by clarifying whether a high‐burden tumor sits within an expected tobacco‐associated molecular context or arises through other mechanisms.

### 4.2. Recurrent Driver Architecture and Downstream Multiomics States

The driver‐level analyses recapitulated well‐established but still clinically relevant exposure‐linked molecular patterns. TP53 and KRAS were enriched in current smokers, whereas EGFR mutations were concentrated in lifelong never‐smokers [29–31]. These contrasts are precisely the kinds of variant‐to‐biomarker transitions that matter in practice because they determine which biomarkers are plausible, which assays are most informative, and how clinicians should interpret composite molecular results. In this setting, TP53 is best viewed as a smoking‐associated driver‐level correlate that also tracks with high mutational burden; the present design cannot separate these linked interpretations.

The transcriptomic and proteomic screens further showed that high‐TMB tumors occupy a distinct downstream state characterized by cell‐cycle, signaling, DNA‐repair, and immune‐related signals, including higher Cyclin B1, MSH6, and CD274 protein abundance [32–34]. This multilayered structure supports a broader interpretation of TMB as a systems biomarker embedded in downstream functional biology rather than a stand‐alone scalar quantity. These multiomics results should not be read as causal downstream consequences of high TMB; they may also reflect smoking‐linked biology, immune or cell‐cycle context, or unmeasured tumor states.

### 4.3. Implications for Multiomics Implementation and Clinical Translation

Two translational lessons emerge. First, parsimonious models that combine clinical variables with recurrent driver alterations may offer the best balance of discrimination, interpretability, and deployability when sample size is limited. In the present cohort, that model outperformed the broader multiomics classifier. Second, broader multiomics integration still added value by revealing biologically coherent states and by generating an integrated score associated with survival. In other words, the broadest model was not the most discriminative, but it remained informative for biological contextualization and downstream risk stratification. The present results do not establish a clinically deployable model: External validation, calibration, prospective workflow testing, and evaluation against treatment‐response data would be required before clinical use.

These findings are relevant to the broader variant‐to‐biomarker agenda because real‐world implementation depends not only on assay performance but also on transparent exposure ascertainment, model interpretability, and transportability across populations and institutions [35–37]. Smoking history is often incompletely captured in clinical workflows, and public reference datasets can underrepresent some racial, socioeconomic, and treatment contexts [38–40]. Exposure‐aware biomarker translation therefore requires both analytic rigor and careful attention to dataset provenance, workflow standardization, and equity in validation. Accordingly, the findings are relevant to biomarker interpretation and hypothesis generation, but they should not be used for direct immunotherapy decision‐making in the absence of treatment‐response analyses.

### 4.4. Strengths and Limitations

Strengths of this study include the use of a public, well‐characterized multiomics cohort; a prespecified environmental exposure of major etiologic relevance; consistent adjusted regression modeling; FDR‐controlled differential analyses; and additional spline, predictive, and survival analyses that extend a standard secondary‐analysis framework. The study also has limitations. This was an observational secondary analysis of public data and causal inferences are not warranted. Smoking exposure was based on recorded clinical annotations rather than biomarker‐confirmed exposure. The TCGA cohort is enriched for surgically characterized tumors and is not a population‐based sample. Multiomics sample size was smaller than the full clinical cohort, and no independent external validation cohort was available. Finally, the prognostic analyses were exploratory and should be interpreted accordingly. In particular, the survival analyses should be viewed as hypothesis‐generating because public TCGA data do not fully capture systemic therapies, immunotherapy exposure, comorbidities, and other prognostic variables that may influence overall survival.

## 5. Conclusions

In TCGA lung adenocarcinoma, cumulative tobacco burden was associated with high TMB, tobacco‐linked substitution patterns, and recurrent driver architecture, whereas high‐TMB tumors displayed coherent transcriptomic and proteomic states. A driver‐augmented model provided the strongest internal discrimination of high TMB, whereas the broader integrated multiomics score retained survival relevance. These results support exposure‐aware, interpretable multiomics biomarker development for clinical translation. They remain hypothesis‐generating until externally validated in cohorts with standardized exposure ascertainment, clinically calibrated TMB assays, treatment data, and outcome annotation.

## Author Contributions

Conceptualization: Jin Wei, Yang Conggao, Ruan Hongjia, Zhu Bingqiang, and Qin Yangsong; methodology: Jin Wei, Yang Conggao, Ruan Hongjia, Zhu Bingqiang, and Qin Yangsong; validation: Jin Wei, Yang Conggao, Ruan Hongjia, Zhu Bingqiang, and Qin Yangsong; formal analysis: Jin Wei, Yang Conggao, Ruan Hongjia, Zhu Bingqiang, and Qin Yangsong; investigation: Jin Wei, Yang Conggao, Ruan Hongjia, Zhu Bingqiang, and Qin Yangsong; resources: Jin Wei, Yang Conggao, Ruan Hongjia, Zhu Bingqiang, and Qin Yangsong; data curation: Jin Wei, Yang Conggao, Ruan Hongjia, Zhu Bingqiang, and Qin Yangsong; writing—original draft: Jin Wei, Yang Conggao, Ruan Hongjia, Zhu Bingqiang, and Qin Yangsong; writing—review and editing: Jin Wei, Yang Conggao, Ruan Hongjia, Zhu Bingqiang, and Qin Yangsong; project administration: Jin Wei, Yang Conggao, Ruan Hongjia, Zhu Bingqiang, and Qin Yangsong. All authors of this paper have reviewed the manuscript of this version and agree to its submission.

## Funding

No funding was received for this manuscript.

## Ethics Statement

This study was reviewed by the Ethics Committee of Kunming University of Science and Technology, which determined that, since the study utilized only publicly available deidentified data, it did not require approval.

## Consent

The authors have nothing to report.

## Conflicts of Interest

The authors declare no conflicts of interest.

## Supporting information


**Supporting Information** Additional supporting information can be found online in the Supporting Information section. Tables S1, S2, S3, S4, S5, and S6 and Figures S1, S2, S3, and S4 are submitted as a separate Supporting Information. The Supporting Information tables report endpoint‐specific pack‐year associations, recurrent‐driver alterations by smoking category, transcriptomic and proteomic features associated with high TMB, cross‐validated model performance, and overall‐survival models. The Supporting Information figures provide the integrated multiomics heatmap, receiver‐operating characteristic curves, Kaplan–Meier curves, and the analytic cohort flow diagram.

## Data Availability

The data that support the findings of this study are available from the corresponding author upon reasonable request.
