# Supplementary material for: From Exposure to Biomarker: Cumulative Tobacco Burden and Integrated Multiomics Signatures of High Tumor Mutational Burden in Lung Adenocarcinoma—A Secondary Analysis of the Cancer Genome Atlas
Source: Hum Mutat. 2026 Jul 8;2026:1906706. doi: 10.1155/humu/1906706 (PMC13346775; doi:10.1155/humu/1906706)
Supplement: Supplementary file 1 — Supporting Information Additional supporting information can be found online in the Supporting Information section. Tables S1, S2, S3, S4, S5, and S6 and Figures S1, S2, S3, and S4 are submitted as a separate Supporting Information. The Supporting Information tables report endpoint‐specific pack‐year associations, recurrent‐driver alterations by smoking category, transcriptomic and proteomic features associated with high TMB, cross‐validated model performance, and overall‐survival models. The Supporting Information figures provide the integrated multiomics heatmap, receiver‐operating characteristic curves, Kaplan–Meier curves, and the analytic cohort flow diagram. [file HUMU-2026-1906706-s001.zip › Supplementary Table 1-6 and Supplemtary Figure 1-4/Supplementary Table 1-6.zip!/Supplementary Table 1-6/Supplementary Table 5.docx]

**SUPPLEMENTARY TABLE 5.** Cross-validated performance of models for identifying high TMB

| **Model** | **n** | **Features** | **AUROC (95% CI)** | **Delta vs clinical** | **Predictor set** |
| --- | --- | --- | --- | --- | --- |
| Clinical | 250 | 6 | 0.611 (0.528, 0.685) | -- | Pack-years + demographic/stage covariates |
| Clinical + variants | 250 | 16 | 0.772 (0.707, 0.831) | 0.162 (0.078, 0.247) | Clinical + recurrent driver mutations |
| Clinical + variants + multi-omics | 250 | 101 | 0.744 (0.675, 0.807) | 0.133 (0.045, 0.224) | Variant model + 60 transcripts + 25 proteins with foldwise selection |
| Note: Performance metrics are based on five-fold out-of-fold predictions. AUROC confidence intervals and model-difference confidence intervals were obtained by bootstrap resampling. | | | | | |
